# *ODF4*, *MAGEA3*, and *MAGEB4*: Potential Biomarkers in Patients with Transitional Cell Carcinoma

**DOI:** 10.22034/ibj.22.3.160

**Published:** 2018-05

**Authors:** Mandana Afsharpad, Mohammad Reza Nowroozi, Mohsen Ayati, Mojtaba Saffari, Saeed Nemati, Elham Mohebbi, Leila Nekoohesh, Kazem Zendehdel, Mohammad Hossein Modarressi

**Affiliations:** 1 Cancer Research Center, Cancer Institute of Iran, Tehran University of Medical Sciences, Tehran, Iran; 2 Uro Oncology Research Center, Tehran University of Medical Sciences, Tehran, Iran; 3 Department of Epidemiology and Biostatistics, School of Public Health, Tehran University of Medical Sciences, Tehran, Iran; 4 Department of Medical Biotechnology, School of Advanced Technologies in Medicine, International Campus, Tehran University of Medical Sciences, Tehran, Iran; 5 Cancer Biology Research Center, Cancer Institute of Iran, Tehran University of Medical Sciences, Tehran, Iran; 6 Department of Molecular Medicine, School of Advanced Medical Technologies, Tehran University of Medical Sciences, Tehran, Iran

**Keywords:** Transitional cell carcinoma, Clinical markers, Cancer testis antigen, Human outer dense fiber of sperm tails 4 protein

## Abstract

**Background:**

This study aimed to evaluate the diagnostic value of outer dense fiber 4 (*ODF4*), melanoma-associated antigen A3 (*MAGEA3*), and *MAGEAB4* mRNAs in transitional cell carcinoma (TCC), using a small amount of cell reverse transcriptase-polymerase chain reaction (RT-PCR) on urinary exfoliated cells.

**Methods:**

We recruited a total of 105 suspected TCC patients and 54 sex- and age-matched non-TCC controls. The candidates’ genetic expression patterns were investigated with RT-PCR, while reverse transcription quantitative PCR was applied to quantify and compare each mRNA level between cases and control groups.

**Results:**

The sensitivity of *ODF4*, *MAGEA3*, and *MAGEAB4* RT-PCR was 54.8%, 63%, and 53.4%, whereas the specificity was 73.7%, 86%, and 94.7%, respectively. Combining *ODF4*, *MAGEA3*, and *MAGEAB4* RT-PCR offered a relatively higher sensitivity (83.6%).

**Conclusion:**

RT-PCR with *ODF4*, *MAGEA3*, and *MAGEAB4* on urinary exfoliated cells could provide clinicians with a promising method to improve TCC diagnosis, especially in the case of gross hematuria and catheterization. The method used here is non-invasive, simple and convenient, and unlike cytology, it does not rely directly on expert professional opinions. These features can be of particular importance to the management of TCC patients in whom regular and lifelong surveillance is required.

## INTRODUCTION

Transitional cell carcinoma (TCC) is the most prevalent male urinary tract malignancy[1]. Its high propensity to recurrence (50-80%) leads to the need for lifelong, costly invasive surveillance[2,3]. Cystoscopy, with its high accuracy and appropriate sensitivity rate, remains the gold standard among diagnostic and surveillance procedures, but still has some complications. The main defects include high follow-up costs, inter/intra personal variability in results, discomfort, invasiveness, and its limitation for detecting early-stage tumors, and those located beyond its visible range. Urine cytology, on the other hand, lacks adequate sensitivity, especially in the case of atypical urothelial cells originating from low-grade lesions. Therefore, new non-invasive or less invasive clinical diagnostic approaches are needed.

The specific mRNA signature of epithelial cells of cancerous tissue origin might clarify the proper strategy for the non-invasive diagnosis of bladder cancer[4]. Urine, which can be collected non-invasively, is in direct contact with bladder tumor cells. Urinary tract epithelial turnover is normally low, but it shows an increased vigor under abnormal malignant states such as bladder cancer[5].

Despite the fact that, up until the present, a number of urine-based biomarkers have been introduced by intense ongoing investigations on TCC, none has been proved capable of supplanting cystoscopy and urine cytology, generally due to their limited specificity[6]. Among tumor-associated antigens, cancer testis antigens (CTAs) are potential attractive biomarkers for tumor specific diagnostic tests because of their wide expression pattern in cancer tissues and restricted presentation in normal tissues.

In our previous case control study (unpublished data), the expression of 16 known candidate CTAs (*MAGEA3*, *MAGEB4*, *BRDT*, *ACRBP*, *TAF7L*, *PASD1*, *TSGA10*, *PIWIL2*, *OIP5*, *AKAP4*, *NUF2*, *MAEL*, *TEX101*, *SPATA19*, *ODF3*, and *ODF4* [outer dense fiber 4]) and seven testis restricted/selective genes (*DDX4*, *DAZ1*-*4*, *POU5F1*, *ACTL7A*, *AURKC*, *CGB2*, and *PLCZ1*) predominantly expressed in the testes were evaluated in urinary exfoliated cells. In addition, the cancerous tissues of clinical/pathological TCC patients, with tumor-free matched, adjusted bladder tissue specimens, were analyzed as controls. Among all studied candidate genes, only *ODF4*, melanoma-associated antigen (*MAGE*) *A3*, and *MAGEB4* mRNA were detectable in more than 50% of both TCC tissues and urinary exfoliated cells and in less than 20% of tumor-free matched, adjusted bladder tissues. Based on those preliminary results, we have hypothesized that the mRNA detection of these candidate genes in urinary exfoliated cells would be applicable for the clinical diagnosis and surveillance of bladder carcinoma. The aim of the present study was to evaluate the diagnostic efficacy of *ODF4*, *MAGEA3*, and *MAGEB4* mRNA in urinary exfoliated cells.

## MATERIALS AND METHODS

### Clinical diagnosis, patients, and samples

A total of 105 patients with a history or clinical suspicion of TCC was recruited into this study as the subjects. The cases were referred to the Department of Urology, Imam Khomeini Hospital Complex (Tehran University of Medical Sciences, Tehran, Iran) from March 2015 to June 2016. The sample size was calculated based on Malhotra *et al*.[7]. The clinical suspicion of TCC in our study was defined as: subjects over 35 years old with positive or inconclusive cytology for TCC, exposed to known and potential risk factors for TCC (including cigarette smoking, opium using, and occupational exposure to aromatic amines) with intermittent painless gross hematuria with clot passage or two or more microscopic hematuria episodes (defined as five or more red cells in urine per high power field at different clinical labs). The control group consisted of 57 sex- and age-matched non-TCC subjects, including healthy volunteers (n = 20) and patients under clinical suspicion of having benign genitourinary disease such as bladder stone, urinary tract infection, benign prostatic hyperplasia (BPH), and obstructive uropathy (Table 1). None of the patients had received prior cytotoxic or radiation therapy during the previous two years.

**Table 1:** Overall prevalence of case and control groups based on the clinical diagnosis along with the prevalence of gross hematuria, urine collection through a catheter, and inconclusive cytology results in each category

Sample type	Clinical diagnosis (%)	Gross hematuria (%)	Catheter (%)	Inconclusive cytology
Low-grade TCC	35 (26.9)	3 (8.5)		10 (28.6)
High-grade TCC	38 (29.2)	8 (21.0)	3 (7.9)	12 (31.6)
Normal	20 (15.4)			2 (10.0)
Benign prostatic hyperplasia	14 (10.8)	1 (7.1)	4 (28.6)	3 (21.4)
Bladder stone	16 (12.3)	2 (12.5)	10 (62.5)	2 (12.5)
Obstructive uropathy	7 (5.4)	2 (28.6)	3 (42.9)	2 (28.6)

TCC, transitional cell carcinoma

The first morning complete fresh voided urine samples were collected and coded randomly by an independent assistant nurse before any clinical and surgical intervention. Invasive urine collection methods such as catheterization and conditions like gross hematuria were considered as known interfering factors in the accuracy of some urinary biomarker tests[8]. To evaluate possible complications in reverse transcription polymerase chain reaction (RT-PCR) diagnostic accuracy, the clinical data of all cases and controls, including urine specimen collection methods (voided urine or urine obtained by catheterization) and hematuria status (gross or microscopic hematuria) were recorded (Table 1).

All urine samples were stored at 4 °C for a maximum of four hours. Specimens were then centrifuged at 800 ×g at 4 °C for 10 minutes (Hettich Universal 320R, Tuttlingen, Germany), in 25-50-mL volumes using 50-mL falcon tubes. The cell pellets were resuspended in 1 mL 1× PBS and transferred to 1.5-µL polypropylene microtubes. Next, 10 µl of each suspension was microscopically examined, and the cells were counted by an independent cytologist, and the remainder was centrifuged at 800 ×g at 4 °C for 5 minutes (Boeckel, Hamburg, Germany). The supernatant was decanted, and the cell pellets were treated with TRIpure reagent (Roche, Germany). The treated cell pellets were then stored at -80 °C for a maximum of one month before testing. All tumor specimens, obtained by conventional cystoscopy from clinically suspected TCC patients, were examined by a pathologist. Pathologically and/or cytologically the confirmed cases of TCC were classified as positive and divided into the high grade and low grade, based on the histological differentiation degree and the pathologic stage, according to the World Health Organization system classification[9].

All participants received a detailed description of the purpose and the procedures of this investigation and signed an informed consent. The study protocol was approved by the Ethics Committee of Tehran University of Medical Sciences.

### RNA extraction, integrity assessment, and complementary DNA (cDNA) synthesis

Total RNA was isolated from urinary cell pellets by TRIpure (Roche, Germany) treatment, according to the manufacturer’s protocol. The quantity and purity of each RNA sample were measured with a NanoDropND 2000 Spectrophotometer (Thermo Fisher Scientific Inc., Wilmington, DE, USA), while sample integrity was confirmed with electrophoresis on 1.0% agarose gel (UltraPure™ Agarose, Invitrogen, USA) in 2.2 M formaldehyde. High-quality RNA samples that showed no degradation were stored at -80 °C for a maximum of one month before further analyses.

A total of 500 ng of RNA elution in 10-µL total volumes was primed with oligo-dT and subjected to cDNA synthesis according to the protocol provided by the manufacturer (PrimeScript™ RT reagent kit, TaKaRa, Japan). To verify mRNA existence and DNA contamination, PCR amplification of ribosomal protein S13 was carried out. Briefly, an intron spanning primer pair: forward: 5’ AAGTACGTTTTG TGACAGGCA 3’ and reverse: 5’ GGTGAATCCGG CTCTCTATTAG 3’[10] was used under an initial heating at 95 °C for 5 min and 30 cycles of amplification, followed by a final extension of 8 min at 72 °C.

### RT-PCR amplification

The mRNA expression of the candidate genes was investigated with RT-PCR using oligonucleotide primer pairs recorded in Table 2. Amplifications were performed in an ABI thermal cycler (Applied Biosystems, USA) by adding 2 μl of 1:5 diluted cDNA of each sample to 10 µl PCR master mix (Ampliqon, Denmark), 8 µl nuclease free water, and 0.5 µl each forward/reverse primer (10 pm). All PCR reactions were conducted under 3-min initial denaturation at 95 °C, followed by 35 cycles of amplification and final extension at 72 °C for 8 min. Cases with a detectable PCR product were considered positive. The intensities of PCR products were heterogeneous on SYBR-stained gels; therefore, semiquantitative PCR analysis was conducted by classifying RT-PCR products from 0 (negative) to 4 (strongest signal). Cases with fainted bands were scored positive only if the result was reproducible with repeated RT-PCR. The RT-PCR scores of each case were further compared to reverse transcription quantitative PCR (RT-qPCR) cycle of quantification (Cq) values, and any cases showing great disagreement were repeated to reach a reasonable agreement.

**Table 2:** Primer characteristics of selected candidate genes

CT identifier*	Gene symbol	Primer sequence (5´→3´)	Ref.	Gene title	Amplicon length (bp)	Intron spanning	Gene ID
CT1	*MAGEA3*	F:GTCGTCGGAAATTGGCAGTAT R:TGGGGTCCACTTCCATCAG	[23]	Melanoma antigen family *A3*	100	No	4102
CT3	*MAGEB4*	F:ACGAAGATGTTAGTGCAGTTCC R:GTGCGCTGAGAGACTTTCC		MAGE family member *B4*	137	No	4115
CT136	*ODF4*	F:GCTTATCCTATACTTCACCTGCG R:GCCAGGAGTTCAGAAAAGATTACAC	[24]	Outer dense fiber of sperm tails 4	227	Yes	146852

* Cancer-testis antigen identifier according to CTDatabase (http://www.cta.lncc.br/index.php)

### RT-qPCR

Quantitative PCR of all samples and controls was performed in triplicate, in order to control intra-assay reproducibility. The expression of housekeeping gene, HSP90AB1 (heat shock protein 90-kDa alpha cytosolic, class B member 1), was used to normalize the PCR reaction, as it was previously selected as the two most stable reference gene among tested candidate by BestKeeper, geNorm, and NormFinder[11]. HSP90AB1 has also been identified as the most stable genes reference gene in a previous study on bladder carcinoma tissue[12].

Amplification was carried out in a total volume of 20 µL containing 1× SYBR®Premix Ex Taq™II (TliRNaseH Plus) kit (TaKaRa, Japan), 2.5 pM each sense and antisense primers plus 1 µL cDNA template derived from 500 ng RNA in 0.1 ml PCR strip tubes (QIAGEN GmbH, Germany). Negative controls (no RNA template, no reverse transcriptase, and no cDNA template samples) were also included in each run. The highest amplification, product specificity, and absence of primer dimer were achieved by performing each reaction on the QiagenRotor Gene™ 6000 (Corbett Research, Mortlake, Australia) under 30-s enzyme activation at 95 ºC, followed by 45 cycles of denaturation at 95 ºC for 5 s, annealing at 58 ºC for 15 s, and extension at 72 ºC for 20 s. Fluorescence acquisition on channel green was set in the last step of each cycle, and all runs were stopped with a melting curve analysis by raising the temperature from 65 to 95 °C at 1 °C per 5 s. The PCR amplification accuracy of the candidate genes was assessed with melt curve analyses and agarose gel electrophoresis of the first run. Samples with non-specific products in the melting curve analyses were excluded from further analysis. Amplification plots, melting curves, and random agarose gel electrophoresis were also used to check RNA, cDNA, and DNA contamination in each qPCR run. All qPCR experiments showing a Cq less than 40 in any negative control were repeated.

The inter-assay reproducibility of each primer pair was tested through running identical samples in three separate runs on three different days. The mean Cq values of each triplicate set were applied for further analysis, and values of 40 and above were scored negative. The qPCR amplification efficiency (E) and correlation coefficient squared (R^2^) of all primers were previously calculated to ensure the reaction efficiency concordance[11].

### Statistical analysis

Statistical analyses were performed using Stata version 14.1 (STATA Corp. Inc., College Station, TX, USA). To compare the CTA RT-PCR with the gold standard, the Pearson’s chi-square test and the Fisher’s exact test were applied, with the statistically significant level of *p* < 0.05. To compare the CTA RT-PCR performance versus cytology, both single and double test performance indicators, including sensitivity and specificity, positive predictive value, negative predictive value, positive likelihood ratio, negative likelihood ratio, ROC area, and diagnostic odds ratio (DOR) were calculated and compared with the cytoscopy as the gold standard, using diagt command. Furthermore, both single and double test performance indicators of RT-PCR performance versus cytology were calculated separately for urine specimens collected through catheterization and with gross hematuria to evaluate any possible complications in RT-PCR diagnostic accuracy.

For quantitative relative analysis between different sample groups, raw fluorescence data obtained by Rotor Gene Q series software 2.1.0 were saved as LinReg export format (*.csv) and subsequently loaded into the LinRegPCR software (version 2015.3). The averages of cycle threshold (Ct) values obtained from at least two of each triplicate were input directly in Relative expression software tool (REST^©^) 2009 (QIAGEN Group, Hilden, Germany) for statistical analysis of relative expression results in real-time PCR[13].

## RESULTS

### Patient characteristics

Among 105 patients, 30 were excluded due to either the lack of TCC pathological confirmation or the failure of RT-PCR amplification. Further analysis was performed on 73 patients with pathologically diagnosed positive incident or recurrent TCC (69 males and 4 females). The mean (±SD) age for cases and controls were 63.97 (±12.56), and 63.84 (±15.34), respectively, ranging from 40 to 88 years. Our 57 TCC free matched controls included 20 healthy volunteers (in terms of urology-associated issues) along with individuals diagnosed as having bladder stones (n = 16), BPH (n = 14), and obstructive uropathy (n = 7), as shown in Table 1.

### Cytology

**Fig. 1:**
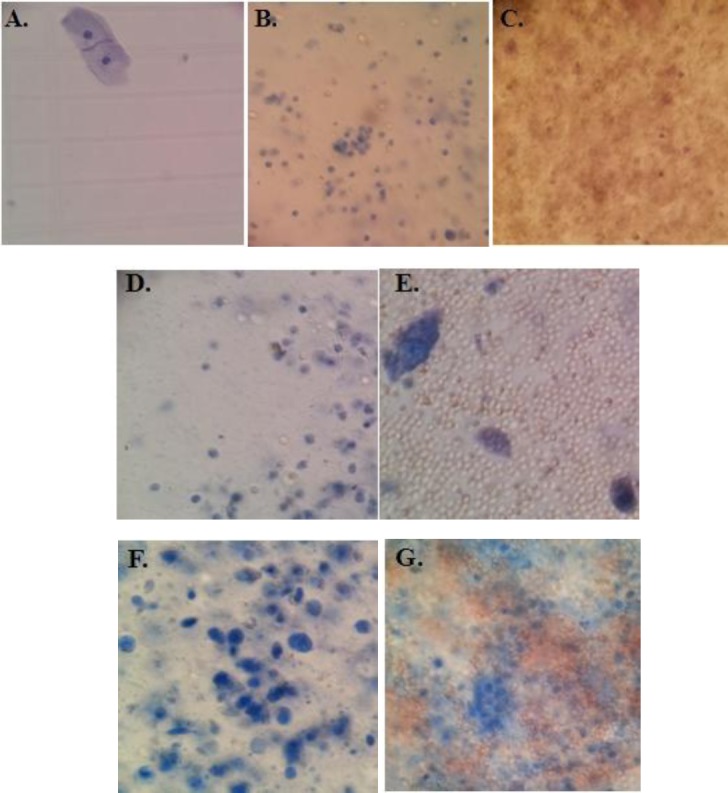
Representative microscopy image of urinary exfoliated cells isolated from a male aged 55-60 (Trypan blue staining). Cells were isolated from the first morning urine of (A) healthy volunteer, (B) benign prostatic hyperplasia (BPH) patient, (C) BPH patient with gross hematuria, (D) bladder stone patient, (E) bladder stone patient with gross hematuria, (F) transitional cell carcinoma (TCC) patient, and (G) TCC patient with gross hematuria. The microscopic patterns of urinary exfoliated cells associated with obstructive uropathy were mostly similar to those from bladder stone patients. The majority of samples followed the same microscopic pattern. As illustrated in the cell patterns here, remarkable changes were observed in the total number of epithelial cells between malignant and non-malignant specimens.

All patients and controls were categorized into positive, negative, and inconclusive based on the cytology reports. The overall positive results of the cytology test among 73 malignant cases comprised only 67.1%. The frequency of inconclusive cytology resulted in different clinical statuses is indicated in Table 1. There was an approximately 2-4fold increase in the total number of epithelial cells between cancerous specimens in comparison to different non-cancerous urinary exfoliated cells (Fig. 1). Total number of epithelial cells ranged from 1 × 10^4^ to 9 × 10^5^ in the majority of normal complete specimens of the first morning urine. Precise cell counting in most cancerous samples was more challenging due to irregular membrane and pleomorphism.

### Diagnostic value of ODF4, MAGEA3, and MAGE A4 RT-PCR

A significantly higher positive rates of *ODF4*, *MAGEA3*, and *MAGEB4* were revealed in the urinary exfoliated cells of TCC patients compared to the control group (*p* < 0.05). No significant difference was observed in the mRNA expression of *ODF4* and *MAGEB4* between low-grade and high-grade TCC samples (*p* > 0.05), whereas *MAGEA3* showed significantly higher expression levels in high-grade TCC patients in comparison to low grades (*p* < 0.05). *MAGEA3* RT-PCR revealed 63.0% sensitivity, whereas the specificity was 86%. The DOR of *MAGEB4* RT-PCR was the highest among others (20.6 vs. 10.4 and 3.3), and the sensitivity, specificity, and DOR of *ODF4* RT-PCR were 54.8%, 73.7%, and 3.3%, respectively (Table 3). *ODF4* mRNA was detected in 40 (54.8%) TCC cases, 10 (71.4%) BPH controls, and only 5 (25%) normal individuals (Table 4). The false-negative rate was 33 (out of 73) TCC cases, while 15 false-positive results were observed using RT-PCR. The detection rate of *ODF4* RT-PCR was relatively weaker in the case of BPH, whereas *MAGE* genes showed less affective instability in those cases (Table 4).

**Table 3:** Diagnostic test performance indicators scores computed for each gene RT-PCR

Measure	*MAGEA3*		*MAGEB4*		*ODF4*	
		
	Point estimation (%)	95% CI	Point estimation (%)	95% CI	Point estimation (%)	95% CI
Sensitivity	63	50.9, 74.0	53.4	41.4, 65.2	54.8	42.7, 66.5
Specificity	86	74.2, 93.7	94.7	85.4, 98.9	73.7	60.3, 84.5
ROC area	0.745	0.673, 0.817	0.741	0.676, 0.805	0.642	0.561, 0.742
+LR	4.4	2.6, 7.5	10.2	4.6, 22.2	2.0	1.3, 3.2
-LR	0.4	0.3, 0.5	0.4	0.3, 0.6	0.6	0.4, 0.8
DOR	10.4	4.5, 23.7	20.6	7.4, 57.4	3.3	1.6, 7.0
PPV	85.1	77.2, 90.6	92.8	85.5, 96.6	72.6	63.0, 80.5
NPV	64.6	57.6, 71.0	61.5	55.7, 67.0	56.2	48.8, 63.2

+LR, positive likelihood ratio; -LR, negative likelihood ratio; DOR, diagnostic odds ratio; PPV, positive predictive value; NPV, negative predictive value

**Table 4:** Expression rate by clinical status of candidate gene mRNAs using RT-PCR

Sample type	*ODF4* positive (%)	*MAGEA3* positive (%)	*MAGEB4* positive (%)
TCC	40 (54.8)	46 (63)	39 (53.4)
Low-grade TCC	19 (54.3)	20 (57.1)	22 (62.9)
High-grade TCC	20 (52.6)	23 (60.2)	18 (47.4)
Normal	5 (25)	6 (30)	1 (5)
Benign prostatic hyperplasia	10 (71.4)		1 (7.1)
Bladder stone		1 (6.3)	
Obstructive uropathy		1 (14.3)	1 (14.3)

TCC, transitional cell carcinoma

Combining *ODF4*, *MAGEA3*, and *MAGEB4* RT-PCR, as the panel, offered relatively higher sensitivity. The overall sensitivity and specificity of the RT-PCR panel of these three genes were estimated at 83.6% (95% CI: 73.0 and 91.2) and 61.4% (95% CI: 47.6 and 74.0), while they were 70.0% (95% CI: 57.9 and 80.4) and 84.2 (95% CI: 72.1 and 92.25) for cytology, respectively (Table 5). The total estimated DOR for cytology (DOR = 12.4, 95% CI: 5.5 and 28.1) was slightly higher than the RT-PCR panel of our three genes (DOR = 8.0, 95% CI: 3.7 and 17.6); however, the observed difference was not statistically significant (Table 5). Using both *MAGE* genes as the diagnostic panel provided 78.1% sensitivity and 84.2% specificity with a DOR of 19 (Table 5).

**Table 5:** Diagnostic test performance indicators scores calculated for *MAGE* genes RT-PCR panel, our 3 gene RT-PCR panel, and cytology

Measure	*MAGE* genes RT-PCR panel		3 gene RT-PCR panel		Cytology	
		
	Point estimation (%)	95% CI	Point estimation (%)	95% CI	Point estimation (%)	95% CI
Sensitivity	78.1	66.9,86.9	83.6	73.0, 91.2	70.0	57.9, 80.4
Specificity	84.2	72.1,92.5	61.4	47.6, 74.0	84.2	72.1, 92.5
ROC area	0.811	0.744,0.879	0.725	0.648, 0.802	0.771	0.699, 0.843
+LR	4.95	3.17,7.73	2.1	1.8, 2.8	4.4	1.7, 7.1
-LR	0.26	0.179,0.379	0.2	0.1, 0.4	0.3	0.2, 0.4
DOR	19	8.35,43.2	8.0	3.7, 17.6	12.4	5.5, 28.1
PPV	86.3	80.1 90.8	73.4	67.4, 78.6	84.9	77.7, 90.1
NPV	75.1	67.5,81.5	74.6	64.3, 82.7	68.8	61.3, 75.5

+LR, positive likelihood ratio; -LR, negative likelihood ratio; DOR, ROC area and diagnostic odds ratio; PPV, positive predictive value; NPV, negative predictive value

In our data set, cytology was not able to detect positive and negative cases among the majority of patients with gross hematuria, while the estimated sensitivity (81.8, 95% CI: 48.2 and 97.7) and specificity (80.0, 95% CI: 28.4 and 99.5) for our 3 gene RT-PCR panel were quite equal in those samples in comparison to non-gross hematuria cases with no significant difference (Table 6). The study results depicted that among catheterized patients, the 3 gene RT-PCR panel (DOR = 7.2 95% CI: 0.6 and 75.3) performed slightly better than cytology (DOR = 4.6, 95% CI: 0.4 and 44.9). However, no significant difference was observed (Table 7).

**Table 6:** The 3 gene RT-PCR panel Diagnostic test performance indicators scores in total cases to total controls and gross hematuria cases to gross hematuria controls

Measure	3 gene RT-PCR panel (Total cases to total controls)		3 gene RT-PCR panel (Gross hematuria cases to gross hematuria controls)	
	
	Point estimation (%)	95% CI	Point estimation (%)	95% CI
Sensitivity	83.6	83.6	81.8	48.2, 97.7
Specificity	61.4	61.4	80.0	28.4, 99.5
ROC area	0.725	0.725	0.809	0.5, 1.0
+LR	2.1	2.1	4.0	1.2, 13.6
-LR	0.2	0.2	0.2	0.06, 0.8
DOR	8.0	8.0	18.0	1.5, 213
PPV	73.4	73.4	82.8	59.0, 94.1
NPV	74.6	74.6	78.9	51.4, 93.0

+LR, positive likelihood ratio; -LR, negative likelihood ratio; DOR, ROC area and diagnostic odds ratio; PPV, positive predictive value; NPV, negative predictive value

**Table 7:** The 3 gene RT-PCR panel Diagnostic test performance indicators scores versus cytology test in patients whose urine has been collected through catheterization

Measure	3 gene RT-PCR panel		Cytology	
	
	Point estimation (%)	95%CI	Point estimation (%)	95% CI
Sensitivity	75.0	19.4, 99.4	50.0	6.7, 93.2
Specificity	70.6	44.0, 89.7	82.4	56.6, 96.2
ROC area	0.728	0.4, 0.9	0.662	0.3, 0.9
+LR	2.5	0.8, 7.7	2.8	0.6, 13.1
-LR	0.3	0.1, 1.2	0.6	0.2, 1.2
DOR	7.2	0.6, 75.3	4.6	0.4, 44.9
PPV	37.4	16.4, 64.5	39.9	12.6, 75.4
NPV	92.3	77.8, 97.6	87.5	77.1, 93.6

+LR, positive likelihood ratio; -LR, negative likelihood ratio; DOR, ROC area and diagnostic odds ratio; PPV, positive predictive value; NPV, negative predictive value

### RT-qPCR results

**Fig. 2:**
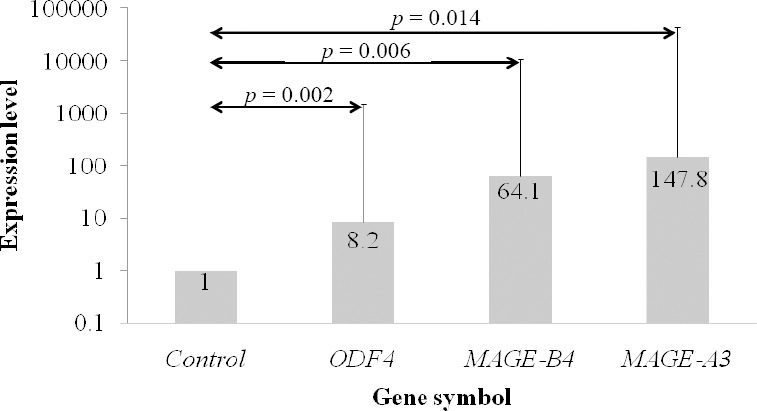
Candidate gene expression levels analyzed with relative expression software tool (REST©) 2009 (QIAGEN Group, Hilden, Germany) and normalized to HSP90AB1. The expression levels of each gene are plotted relative to the control group, and the exact values are illustrated in boxes.

*ODF4*, *MAGEA3*, and *MAGEB4* mRNA expression was measured with RT-qPCR in urinary exfoliated cells isolated from all cases and controls along with one testis positive control sample, using REST^©^ 2009 (QIAGEN Group, Hilden, Germany)[13]. The results were completely in concordance with the simple RT-PCR approach, i.e. *ODF4*, *MAGEA3*, and *MAGEB4* mRNA were detected in 40 (54.8%), 46 (63%), and 39 (53.4%) TCC patients and 15 (26.3%), 8 (14%), and 3 (5.3%) non-TCC individuals, respectively. All cases that were scored 0 (negative) by RT-PCR showed RT-qPCR non-specific products or Cq values of 40 and above. The scores of 4, 3, 2, and 1 for HSP90AB1, *MAGEA3*, *MAGEB4*, and RT-PCR corresponded to Cq values of less than 24, 25 27.99, 28 29.99, and over 30 (and less than 40), respectively. The minimum Cq values of the *ODF4* was 27.40, which corresponded to RT-qPCR score of 4 along with Cq values up to 28.99, while Cq values of 29 30.99, 31 32.99, and 33 34.99 corresponded to RT-qPCR score of 3, 2, and 1, respectively. Significant up-regulation was detected in the expression level of the cases in comparison to the control group (up to 8.2fold in *ODF4*, 147.8fold in *MAGEA3*, and 64.1fold in *MAGEB4*; *p* < 0.05; Fig. 2).

## DISCUSSION

In the present study, the mRNA expression status of *ODF4*, *MAGEA3*, and *MAGEB4* genes was evaluated in urinary exfoliated cells of TCC patients in comparison to non-TCC individuals. In addition, the association of the expression status of these genes with TCC was also investigated. The results indicated that the expression analysis of these three genes can be considered as a promising diagnostic and screening biomarker, especially in the case of patients with gross hematuria and catheterization. The panel derived by combining these three genes can provide up to 83.6% sensitivity, which is higher than that obtained by cytology (70.0%; Table 6).

We found our 3 gene RT-PCR panel easier and more practical than similar current biomarkers regarding the methodology and analysis. The Cxbladder Detect (Pacific Edge, Hummelstown, PA, USA), a multigene urine test, reported 85% sensitivity[14]. This test was designed based on the significant increase of IGFBP5, HOXA13, MDK, CDK1, and CXCR2 mRNAs in voided urine using RT-qPCR. According to a meta analysis study, the pooled sensitivity for urine survivin tests was found to be 77%[15]. The assessment of AURKA mRNA over expression using RT-qPCR provided 84% sensitivity[16]. The panel introduced here showed a higher sensitivity in our dataset, and was also much easier, since we used simple RT-PCR instead of RT-qPCR. Moreover, the result we obtained with RT-PCR using urinary exfoliated cells was completely in concordance with the RT-qPCR result. It is also worth mentioning that the isolation of mRNAs from cells is much more practical than detecting free urine mRNAs, owing to the total isolated mRNA amount and less stability of mRNAs in the urine environment.

Our 3 gene panel method might be helpful for the diagnosis of early-stage TCC, since no significant difference was observed in the mRNA expression of *ODF4* and *MAGEB4* between low grade and high grade TCC groups (*p > 0.05*). This point assumes importance considering that 70% to 80% of TCC patients suffer from low grade tumors with a recurrence rate of up to 70%[17]. Conversely, *ODF4* and *MAGEB4* may not be beneficial as prognostic biomarkers, though *MAGEA3* might offer an appropriate prognostic value. An association between *MAGEA3* expression and both the stage and grade of TCC has previously been shown[18]. Therefore, it appears that they both could be used as the early-stage diagnostic and prognostic factor when using together.

Through evaluating the expression status of *ODF4*, *MAGEA3*, and *MAGEB4* RT-PCR in benign urothelial diseases, we found the false-positive rate of 15 (26.3%), 8 (14%), and 3 (5.3%) respectively. Most of the *ODF4* false-positive cases were BPH patients. As described previously, MAGE genes were occasionally expressed in the presence of chronic inflammatory reactions[19]. Expression of *ODF4* at the mRNA level in the majority of BPH patients can become a major obstacle for detecting TCC, since TCC is generally a disease observed in middle-aged and elderly populations with a median age of 70 years at the time of diagnosis. This result matches the height of the age for BPH prevalence, as BPH shows an increased incidence from 1.2% in men 40-49 years of age to 36% in those >70 years[20]. The fact that 97.3% of the patients in our dataset were over 40 years old could also bring to mind that *ODF4* expression pattern at mRNA level was in response to the BPH rather than the TCC status. The frequent expression of *ODF4* within TCC tumor tissues and its correlation with the expression data from urine correctly negates that the expression pattern of *ODF4* at mRNA level was in response to the BPH, but not to the TCC status (unpublished data). The significant change in the expression level of *ODF4* between the TCC cases and the control groups (*p* < 0.05) is also in opposition to this hypothesis.

Taking the above data together, it might appear that our proposed 3 gene RT-PCR panel could still not provide sufficient specificity to entirely supplant cystoscopy. Although the sensitivity of the *MAGE* gene RT-PCR panel is less than our 3 gene RT-PCR panel (78.1% vs. 83.6%), it offers higher specificity (84.2% vs. 61.4%) and DOR (19 vs. 8; Table 5). Finding more specific substitutes for *ODF4* would be of practical importance, since it alone causes the majority of false-negative findings (DOR = 3.3 in comparison to 10.4 and 20.6 for *MAGEA3* and *MAGEB4*, respectively). The DOR is an effective measure of diagnostic test, being defined as the ratio of the odds of the test being positive if the subject has a disease relative to the odds of the test being positive if the subject does not have the disease. It is independent of the prevalence and range from 0 to infinity. Higher DOR values represent the better performance of diagnostic tests and are only obtained by higher values of sensitivity and specificity.

Considering the invasiveness and complications of cystoscopy as the gold standard test, RT-PCR could easily provide a convenient alternative option in the daily clinical setting, as a primary detection technique in suspected urothelial cancer patients. The method we used in the present study is quite close to that proposed by Chiu *et al*. in 2002[21]. We found our 3 gene RT-PCR panel to be non-invasive, simple and convenient. Unlike cytology, it does not rely directly on expert professional opinions. We therefore believe that it could be considered as a substitute for cytology and other current adjutant diagnostic tools. Our data also indicated that RT-PCR may be applied as a TCC diagnostic approach for patients with gross hematuria and in the case of urine collection through catheterization. It is also compatible with a small number of exfoliated cells, since PCR primers can detect a limited number of cancer cells in urinary exfoliated cells. Consequently, this method does not require a large amount of urine. Another important point that needs to be considered is the remarkable changes in the total number of epithelial cells between the malignant and non-malignant states (Fig. 1). Our previous study also indicated a significant increase in RNA concentrations of urinary exfoliated cells isolated from TCC patients in comparison with non-TCC individuals, which was in accordance with the microscopic image analysis[11]. The remarkable changes in the total number of epithelial cells between the malignant and non-malignant states would be of considerable benefit, further proving that the specific antigen applicable for this method does not need to be expressed in large proportions.

In addition to the diagnostic potentials, CTAs are also considered attractive for an immunotherapy and vaccine target because of their restricted expression pattern and highly immunogenic nature. Although clinical applications of active-targeting *MAGE* family antigens have recently been questioned due to cross reactivity between testis restricted and non-testis restricted *MAGE* antigens[22], *ODF4* might still be a promising target for TCC immunotherapy research. Complementary protein analysis would be needed for defining and monitoring the functional ability of the actual *ODF4* protein in malignant tissues.

*ODF4*, *MAGEA3*, and *MAGEB4* RT-PCR, using urinary exfoliated cells, illustrated appropriate sensitivity and specificity in our dataset, which can be used as putative TCC biomarkers in a multi-biomarker panel form or as an adjunct to conventional diagnostics. Considering the increasing number of TCC cases in Iran, especially in the southeastern part of Kerman Province, the set-up of the 3 gene panel discussed in this study would have a significant clinical impact, since it is simple, convenient, and non-invasive for daily laboratory use.

Although the sample size we used here was enough to examine the main hypothesis of this study[7], in order to perform detailed sub-analyses and observe other possible existing statistical associations, further investigations are needed on this topic using larger sample sizes, preferably from other ethnicities with different genetic, epigenetic, environmental and lifestyle background. It might also be more helpful to assess the accuracy of this method among a healthy high-risk population as well as in middle-aged to elderly males and heavy smokers, to assess and confirm the value of this method for clinical diagnosis.
